# Ehlers-Danlos Syndrome, Hypermobility Type, Is Linked to Chromosome 8p22-8p21.1 in an Extended Belgian Family

**DOI:** 10.1155/2015/828970

**Published:** 2015-10-04

**Authors:** Delfien Syx, Sofie Symoens, Wouter Steyaert, Anne De Paepe, Paul J. Coucke, Fransiska Malfait

**Affiliations:** Center for Medical Genetics, Ghent University Hospital, 9000 Ghent, Belgium

## Abstract

Joint hypermobility is a common, mostly benign, finding in the general population. In a subset of individuals, however, it causes a range of clinical problems, mainly affecting the musculoskeletal system. Joint hypermobility often appears as a familial trait and is shared by several heritable connective tissue disorders, including the hypermobility subtype of the Ehlers-Danlos syndrome (EDS-HT) or benign joint hypermobility syndrome (BJHS). These hereditary conditions provide unique models for the study of the genetic basis of joint hypermobility. Nevertheless, these studies are largely hampered by the great variability in clinical presentation and the often vague mode of inheritance in many families. Here, we performed a genome-wide linkage scan in a unique three-generation family with an autosomal dominant EDS-HT phenotype and identified a linkage interval on chromosome 8p22-8p21.1, with a maximum two-point LOD score of 4.73. Subsequent whole exome sequencing revealed the presence of a unique missense variant in the *LZTS1* gene, located within the candidate region. Subsequent analysis of 230 EDS-HT/BJHS patients resulted in the identification of three additional rare variants. This is the first reported genome-wide linkage analysis in an EDS-HT family, thereby providing an opportunity to identify a new disease gene for this condition.

## 1. Introduction

Joint hypermobility is a common finding in the general population with epidemiologic studies showing its presence in over 10% of Caucasians. It is more prevalent among Asians and Africans than Caucasians, with women affected more often than men, and it is usually maximal at birth, decreasing with age [1]. Joint hypermobility has a strong genetic component, with female twin studies showing that at least 70% of the variance in phenotype is attributed to genetic factors [2]. Most people do not develop any problems from their loose joints and might even benefit from it (e.g., musicians, dancers, and gymnasts). However, in a subset of individuals, joint hypermobility gives rise to a range of clinical problems that mainly affect the musculoskeletal system. It can be found as a part of some well-defined heritable connective tissue disorders, including the Ehlers-Danlos syndrome (EDS). EDS comprises a clinically and genetically heterogeneous group of heritable connective tissue disorders that are currently classified according to the Villefranche nosology into six subtypes, based on clinical symptoms, inheritance pattern, and the nature of the underlying biochemical and molecular defect(s) [3]. The major clinical manifestations, including joint hypermobility, skin hyperextensibility, and generalized connective tissue fragility, are present to varying degrees in each EDS subtype. In some of these subtypes, mutations have been identified in genes encoding one of the fibrillar collagen proteins (types I, III, and V collagen) or coding for enzymes involved in the biosynthesis of those proteins (*ADAMTS2* and* PLOD1*). The most prominent clinical feature observed in the hypermobility subtype of EDS (EDS-HT, former EDS type III) is generalized joint hypermobility with recurrent dislocations and subluxations, in addition to mild skin hyperextensibility and fragility. The joint hypermobility leads to significant joint instability, chronic debilitating pain, and loss of muscle strength, which ultimately results in important physical handicap and often causes great psychological distress. Although EDS-HT is one of the most prevalent EDS subtypes, it remains a challenging condition, at both the clinical and molecular level. There is currently no clear consensus regarding the exact clinical definition and nosologic delineation of EDS-HT. Although considered one and the same by many authorities, there is still debate whether EDS-HT and benign joint hypermobility syndrome (BJHS), which show important phenotypic overlap, represent the same disorder [4]. Moreover, the molecular basis of these conditions remains at present poorly explored. A major drawback for genetic studies is the variable phenotypic expression and reduced penetrance within and between families, making it often difficult to ascertain whether an individual is affected or not.

Families in which the EDS-HT or BJHS phenotype is transmitted in a clear autosomal dominant fashion provide unique genetic models for the study of genes and molecular pathways that are involved in the pathogenesis of joint hypermobility. However, only a limited number of clinically well-defined large families with EDS-HT or BJHS are available for informative genetic linkage studies. In the past, sporadic defects have been identified in the genes encoding fibrillar types I, III, and V collagen, but in the large majority of EDS-HT patients, molecular studies have not revealed causal defects in any of these genes. Ultrastructural studies in patients with EDS-HT have revealed abnormalities in both morphology and diameter of the collagen fibrils, suggesting that impaired collagen fibrillogenesis plays a central role in its pathogenesis [5]. Tenascin-X (*TNXB*) haploinsufficiency was reported in a small subset (5–10%) of patients with EDS-HT and BJHS [6, 7]. This glycoprotein is involved in the regulation of collagen fibrillogenesis. More recent findings point to a role for other genes in the pathogenesis of rare EDS(-like) conditions, including genes encoding a zinc transporter (*SLC39A13*) [8, 9], a peptidyl-prolyl* cis*-*trans* isomerase (*FKBP14*) [10], and genes involved in the biosynthesis of glycosaminoglycans (*B4GALT7*,* B3GALT6*,* CHST14,* and* DSE*) [11–13]. These observations demonstrate that other noncollagenous molecules involved in the modification, folding, or extracellular assembly of collagens could be causally linked to the pathogenesis of EDS and joint hypermobility.

We present the identification of a new genetic locus for EDS-HT in a large three-generation Belgian family through genome-wide linkage analysis. In addition to sequencing a number of interesting positional candidate genes, we applied whole exome sequencing for two affected individuals in an attempt to unravel the underlying genetic cause within this candidate locus.

## 2. Materials and Methods

### 2.1. Patients

We examined a large three-generation family from Belgian descent with EDS-HT. Affected family members presented generalized joint hypermobility with related musculoskeletal problems, in association with a mild dermal phenotype, including soft skin, mild atrophic scarring, and easy bruising. Thirty-four family members were examined after full informed consent was obtained in accordance with requirements of the Ethics Committee of the Ghent University Hospital. Clinical history was taken from all individuals and clinical examination was performed at two different time points (time 0 and after 24 months) by the corresponding author. In addition, another clinical geneticist examined all individuals independently. Individuals were classified as being “affected” if they fulfilled the Villefranche criteria for EDS-HT [3] and the revised Brighton criteria for BJHS [4] and as “unaffected” if they did not fulfill these criteria. Individuals presenting with a milder joint phenotype but with cutaneous manifestations compatible with EDS-HT were classified as “unknown” (Table 1).

Blood and/or skin biopsy specimens were obtained and RNA and genomic DNA (gDNA) were isolated. Total RNA was isolated from cultured skin fibroblasts by Trizol and treated with RNase-free DNase (Life Technologies Europe, Ghent, Belgium). For the conversion to cDNA, Moloney murine leukemia virus reverse transcriptase was used in combination with random hexanucleotide primers (Invitrogen, Life Technologies Europe). gDNA was isolated from fibroblast cultures by the Easy-DNA kit (Invitrogen) or extracted from blood samples using the Qiaquick kit (Qiagen, Hilden, Germany).

### 2.2. Cell Culture and Biochemical Analysis of Collagen Molecules

Fibroblast cultures were established from a skin biopsy from the proband (II.5) and an affected sister (II.6). At confluency, cells were labelled with ^14^C-proline as described earlier [14]. After separation, the gels were processed for fluorography, dried, and exposed to an X-ray film.

### 2.3. Candidate Genes

**Figure 1:**
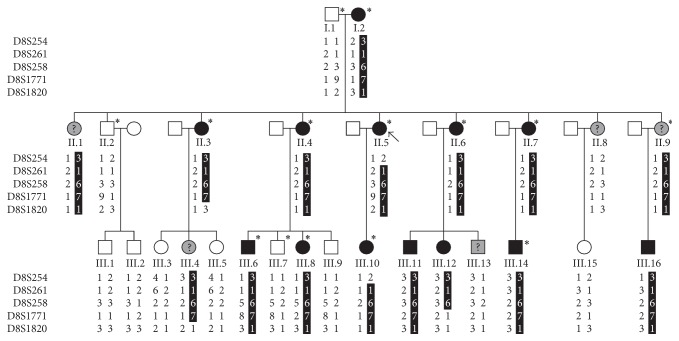
Pedigree of the three-generation Belgian family. The genotypes of 5 markers within and surrounding the linked region on chromosome 8 are depicted. The haplotype cosegregating with the EDS-HT phenotype is indicated with a black bar. An arrow indicates the proband. Individuals included in the initial genome-wide linkage screen are indicated with an asterisk. Affected and unaffected individuals are indicated with a black or white pictogram, respectively, whereas a grey pictogram with a question mark points to a patient for whom the phenotype was not clear (unknown).

As an initial step towards defining the genetic basis of EDS-HT in this family, linkage to a number of candidate genes, including the genes encoding types I, III, and V collagen and tenascin-X (*TNXB*), was tested by use of markers within and surrounding these genes (Supplementary Table 1 in Supplementary Material available online at http://dx.doi.org/10.1155/2015/828970). For the linkage study to these genes, a series of 14 individuals, independently assessed as “affected” (I.2, II.3, II.4, II.5, II.6, II.7, III.6, III.8, III.10, and III.14), “unaffected” (I.1, II.2, and III.7), or “unknown” (II.9) by both investigators, were selected (Figure 1, individuals with an asterisk).

### 2.4. Genome-Wide Linkage Analysis

A genome-wide linkage scan was performed using gDNA from the same 14 individuals (Figure 1, individuals indicated with an asterisk). A set of 400 highly polymorphic microsatellite markers (ABI PRISM Linkage Mapping Set version 2, Applied Biosystems, Foster City, CA, USA) with an average spacing of 10 centimorgans (cM) were analyzed on a capillary sequencer (ABI3100, Applied Biosystems). The data were processed using GeneMapper software (Applied Biosystems). The analysis was performed under the assumption that the condition is inherited as an autosomal dominant trait with a penetrance of 90% at all ages. A disease allele frequency of 0.01 (1/100) and equal recombination fractions in males and females were assumed. Two-point LOD scores were calculated with SuperLink version 1.6.

After the initial assessment of a linked chromosomal region in 14 individuals, gDNA of these individuals and of 13 additional members was further analyzed with four additional microsatellite markers. These markers were chosen from the Marshfield and Généthon genetic map for fine genetic mapping and identifying the critical interval. Order and distance between the markers were obtained from the Généthon genetic database: D8S254, 2.28 cM, D8S261, 4.51 cM, D8S258, 8.5 cM, D8S1771, 5.99 cM, and D8S1820. Haplotypes were constructed, assuming the minimal number of recombination, by tracing the segregation of alleles in the family.

### 2.5. Molecular Analysis and Gene Prioritization

The presence of a causal mutation in one of the genes associated with autosomal dominant forms of EDS was excluded by means of mutation screening of* COL1A1*,* COL1A2*,* COL3A1*,* COL5A1,* and* COL5A2* at cDNA level and of* TNXB* at gDNA level in the proband. Testing for the presence of a* COL5A1* nonfunctional (null-)allele was performed in two affected individuals (II.5 and II.6). In analogy, testing for the presence of a* TNXB* null-allele was performed in the same individuals, using the following polymorphism: c.3482A>G (p.(His1161Arg)) in exon 9.

For mutation analysis of the positional candidate genes* BMP1*,* LOXL2*,* CSGALNACT1,* and* SLC39A14*, gDNA from the proband, one additional affected family member, one unaffected family member, and an unrelated normal control was PCR amplified using intronic primers designed to amplify each exon and adjacent intron-exon boundaries. PCR products were sequenced on the ABI PRISM 3100 automated sequencer (Applied Biosystems). Detected single nucleotide changes were considered a polymorphism either when they were reported in the public databases (dbSNP137 and 1000 Genomes Project), or when they did not segregate with the EDS-HT phenotype within the family.

Gene prioritization was performed with the web-based algorithms Endeavour [15], Suspects [16], G2D [17], GeneDistiller [18], and ToppGene [19], using a training set of 14 EDS-associated genes (*COL1A1*,* COL1A2*,* COL5A1*,* COL5A2*,* COL3A1*,* TNXB*,* PLOD1*,* ADAMTS2*,* CHST14*,* B4GALT7*,* SLC39A13*,* FKBP14*,* B3GALT6,* and* DSE*).

### 2.6. Whole Exome Sequencing

Whole exome sequencing (WES) was performed for two affected individuals (II.5 and II.6) by Aros Applied Biotechnology AS (Aarhus, Denmark). Exome capture was performed using the TruSeq Exome Enrichment kit (Illumina, San Diego, CA, USA) and sequencing was carried out on the Illumina HiSeq 2000 platform with paired-end 100-bp reads. The CLC Genomics workbench version 6.0.4 (CLCBio, Aarhus, Denmark) software was used for read mapping against the human genome reference sequence (NCBI, GRCh37/hg19) followed by duplicate read removal and coverage analysis for all regions enriched with the TruSeq Exome Enrichment kit. Single nucleotide variants and small insertions and deletions were called using the quality-based variant calling and subsequently annotated prior to exporting the resulting variant lists for filtering. The filter strategy focused on retaining heterozygous variants (variant allele frequency between 25% and 75%) shared between both sisters located within the coding sequence or flanking intronic regions (+/−20 bp) of the linkage region. Candidate variants were evaluated using the mutation interpretation software Alamut version 2.2.1 or Alamut HT version 1.1.5 (Interactive Biosoftware, Rouen, France) and segregation analysis was performed for selected variants.

### 2.7. Array Comparative Genomic Hybridization

In order to evaluate copy number alterations within the identified region, array comparative genomic hybridization (aCGH) was performed on individuals II.2, II.9, II.11, II.15, III.2, III.6, III.10, and III.14 (Figure 1) using a custom 8 × 60 K oligonucleotide array (Agilent Technologies, Santa Clara, CA, USA) covering the exonic and intronic portions of the identified candidate region (NCBI, GRCh37.p5/hg19) with a total of 52000 probes. This assay was performed according to the manufacturer's instructions and the results were visualized and analysed using arrayCGHbase [20].

### 2.8. Additional Families

Four additional families were analyzed for linkage to the candidate locus identified in the initial family. These families were examined either by one of the authors (Anne De Paepe or Fransiska Malfait) or by clinical geneticists familiar with the disorder. All families were considered to be affected by EDS-HT, based on the presence of generalized joint hypermobility and related complications of dislocations and chronic pain, in combination with a mild dermal phenotype of soft and/or mildly hyperextensible skin, mild atrophic scarring, and easy bruising.

## 3. Results

### 3.1. Clinical Data

The index patient (Figure 1, II.5) was diagnosed with EDS-HT at young age. She presented severe joint hypermobility since infancy with a life-long history of recurrent subluxations and dislocations of many large and small joints, for example, vertebral bodies, temporomandibular, acromioclavicular, sternoclavicular, glenohumeral, and most other peripheral joints. Since adolescence, she suffered from generalized pain and eventually became wheelchair dependent for long distances at the age of 35 years. She bruised easily after simple trauma and presented delayed wound healing with formation of dilated scars.

After clinical evaluation, 13 individuals were scored as “affected” and 9 as “unaffected.” Affected individuals showed typical features of EDS-HT with joint hypermobility (Beighton score > 5/9), chronic musculoskeletal pain, and, especially in the adults, repetitive dislocations of one or more joints. They all presented easy bruising, and most of them had a soft skin with dilated scars. Many presented striae atrophicae, especially over the thighs and the abdomen. None of them had a marfanoid habitus. Five individuals with mild joint hypermobility and some skin features were scored as “unknown” (II.1, II.8, II.9, III.4, and III.13). A summary of the clinical data is provided in Table 1.

### 3.2. Biochemical Analysis

SDS-PAGE of metabolically labelled types I, III, and V (pro)collagen secreted by and retained in fibroblasts of individual II.5 did not show qualitative or quantitative abnormalities in electrophoretic mobility (data not shown).

### 3.3. Exclusion of Candidate Genes

Segregation analysis for candidate genes* COL1A1*,* COL1A2*,* COL3A1*,* COL5A1*,* COL5A2,* and* TNXB* excluded linkage of the phenotype to one of these genes (Supplementary Table 1). A null-allele test for* COL5A1* and* TNXB* showed that both alleles of these genes were transcribed. Molecular analysis of the* COL1A1*,* COL1A2*,* COL3A1*,* COL5A1,* and* COL5A2* genes at cDNA level and* TNXB* at gDNA level did not reveal a causal mutation. Karyotype of the proband (II.5) was normal (46, XX).

### 3.4. Genomic Linkage Analysis

A systemic genome scan was performed using gDNA of 14 family members in order to identify a predisposition locus for EDS-HT in this family. In the initial genome-wide linkage search, marker D8S258 generated the highest two-point LOD score of 2.5 at theta (*θ*) = 0. No other markers exceeded the threshold LOD score of 2.2, supposing suggestive linkage according to the criteria of Lander and Kruglyak [21]. To confirm and refine this possible predisposition locus, gDNA of these individuals and 13 additional family members was further analysed using four additional microsatellite markers. A haplotype of marker alleles segregates with the disease in all affected individuals (Figure 1). The candidate region was delineated by critical recombination events between D8S254 and D8S261 at the proximal end (telomeric) in individual II.5 and between markers D8S258 and D8S1771 at the distal end (centromeric) in individual III.12. This defined a candidate region of 8.8 Mb, flanked by markers D8S254 and D8S1771 encompassing the cytogenic location 8p22-8p21.1. A maximum two-point LOD score of 4.73 was obtained for D8S258 (Table 2). In order to evaluate the contribution of this locus in other families, the critical interval on chromosome 8 was examined in four additional families with an EDS-HT phenotype. For none of these families linkage of the 8p22-8p21.1 locus with the disease phenotype could be observed (data not shown).

### 3.5. Exclusion of Copy Number Alterations

Copy number profiling was performed using custom aCGH, thereby focusing on the gene-containing regions of the candidate interval. This did not reveal deletions or duplications segregating with the disease phenotype.

### 3.6. Selection and Molecular Screening of Positional Candidate Genes

*In silico* analysis using NCBI MapViewer revealed the presence of 102 known genes within the linkage interval delineated by markers D8S254 and D8S1771 on chromosome 8p22-8p21.1. Of these genes, 20 were indicated as pseudogene, whereas the remaining genes include 69 protein-coding genes, 8 uncharacterized (LOC) genes, three genes encoding long noncoding RNAs, and two microRNAs (Supplementary Figure S1). Within the candidate interval, no known genes associated with EDS were present. The genes located in the identified candidate region were prioritized using multiple web-based prediction tools (Table 3) combined with a literature search. Four functionally interesting candidate genes, including* BMP1* (bone morphogenetic protein-1),* LOXL2* (lysyl oxidase-like 2),* CSGALNACT1* (chondroitin *β*1,4-N-acetylgalactosaminyltransferase), and* SLC39A14* (solute carrier family 39 (zinc transporter), member 14), were selected for Sanger sequencing but no segregating sequence variant that likely explained the connective tissue phenotype was identified. Therefore, we expanded the search for the causal mutation by applying whole exome sequencing.

### 3.7. Whole Exome Sequencing

WES performed for the proband (II.5) and an affected sister (II.6) generated over 132 million reads for each, of which, respectively, 80.4% and 81.2% mapped uniquely against the human reference sequence. Approximately 93% of the enriched TruSeq target regions were covered at least 10-fold in both patients and about 90% of these regions were covered at least 20-fold. An average read depth of 93.7x was achieved for the proband (II.5) and 94.5x for her sister (II.6). Within the identified candidate region, 85.5% of the targeted regions were covered at least 20-fold in both patients whereas 3.4% and 3.6% of these targets were partially (0.6% and 0.9%) or completely (2.8% and 2.7%) uncovered for II.5 and II.6, respectively.

Single nucleotide variants and small insertions and deletions were called using the CLC Genomics workbench. Given the autosomal dominant inheritance pattern, only variants that were heterozygous (with a variant allele frequency between 25% and 75%) in both affected individuals and located within the candidate region on chromosome 8 were considered. This resulted in a total of 300 shared heterozygous variants, which were narrowed down to 11 by selecting variants that result in nonsynonymous amino acid changes, the introduction of a premature termination codon, frameshifts, or (predicted) splice sites changes with an allele frequency below 0.1 in dbSNP137. Comparison with 54 in-house exomes showed that the majority of these variants occurred multiple times in patients suffering from other inherited conditions, thereby omitting these for further analysis. Combined with the exclusion of variants that were predicted to be benign by all four* in silico* prediction programs (SIFT, PolyPhen-2, AlignGVGD, and MutationTaster), this filtering strategy resulted in one unique missense variant p.(His211Gln) located within the* LZTS1* gene, encoding leucine zipper, putative tumor suppressor 1 (previously also reported as* FEZ1*).* In silico* prediction tools indicated that the p.(His211Gln) substitution was predicted to be tolerated by SIFT and AlignGVGD but was considered damaging by both PolyPhen-2 and MutationTaster. The affected amino acid residue is moderately conserved. Sanger sequencing could confirm the presence of this variant in all affected individuals whereas unaffected family members lacked the variant. Subsequently, we assessed the presence of* LZTS1* sequence alterations in 230 probands diagnosed at or referred to our center with EDS-HT or BJHS. This resulted in the identification of three additional variants. The first variant, c.49C>G, p.(His17Asp) was predicted to be damaging by both PolyPhen-2 and MutationTaster and was reported a single time (1 in 13006 alleles, rs368057069) in the NHLBI exome sequencing project exome variant server (http://evs.gs.washington.edu/EVS/). The second variant, c.1585C>T, p.(Arg529Trp) was found in two unrelated individuals and was predicted to be damaging by SIFT, PolyPhen-2, and MutationTaster. The third variant, c.749C>A, p.(Ser250^*∗*^) introduced a premature termination codon in exon 2 and is predicted to result in nonsense mediated mRNA decay (Table 4).

## 4. Discussion

Genetic studies in EDS-HT and BJHS are scarce because they are hampered by several difficulties. First, the diagnosis in an individual is not always obvious. Since joint hypermobility is common in the general population, it is often difficult to make a clear distinction between those individuals at the upper end of the normal “joint mobility spectrum” and those whose increased mobility reflects the presence of a connective tissue disorder. For the vast majority of the EDS-HT patients, the underlying molecular defect remains unknown. Therefore, the diagnosis is entirely based on correct assessment of the clinical phenotype as no pathognomonic, radiographic, biochemical, ultrastructural, or other abnormalities are available to confirm this. The joint hypermobility may decrease or even completely disappear with increasing age. The extra-articular symptoms such as the skin hyperextensibility, abnormal scarring, or other signs of connective tissue fragility may be very subtle. Second, the inheritance pattern in a family is not always straightforward because of variable penetrance and phenotypic expression. Joint hypermobility may be influenced by specific environmental factors, such as hormonal status and degree of activity, and by different, yet to be identified, genetic factors, such as polymorphic genetic determinants in genes encoding proteins of the extracellular matrix. Large families suitable for informative linkage studies are therefore scarce.

This report presents the first successful linkage study in a unique three-generation Belgian family in which EDS-HT segregates with autosomal dominant inheritance pattern. In order to make the clinical assessment in this family as accurate as possible, the same physician (Fransiska Malfait) was responsible for repeated clinical evaluation and history taking. In addition to assessment of joint hypermobility and musculoskeletal problems, a number of clinical signs, indicative of generalized connective tissue fragility, were registered. These were helpful for determining the phenotype in individuals who were ambiguous on the joint phenotype alone. After exclusion of a number of candidate genes, a suggestive candidate locus was identified, which segregates with the EDS-HT phenotype. The highest two-point LOD score of 4.73 was obtained for marker D8S258, highly suggestive for linkage between the phenotype and this locus. A haplotype of marker alleles segregated with the phenotype and critical meiotic recombinants placed the EDS-HT predisposition gene within an 8.8 Mb region on chromosome 8p22-8p21.1. Moreover, the absence of linkage for the identified locus in several other EDS-HT families underscores the genetic heterogeneity of the disorder.

Four of the protein-coding genes located within this region were considered potentially promising candidate genes for EDS-HT.* BMP1* belongs to secreted metalloproteinases and was first described as a procollagen C-proteinase for types I–III procollagen but is also responsible for the cleavage of multiple other substrates (e.g., prolysyl oxidase, prodecorin, and probiglycan). Biallelic defects in* BMP1* were recently described as the cause of a severe recessive variant of the brittle bone disorder Osteogenesis imperfecta [22].* LOXL2* encodes lysyl oxidase-like 2, which belongs to the family of lysyl oxidases and catalyses covalent cross-linking of several fibrillar collagen types and elastin, whereas* CSGALNACT1* encodes a key enzyme for chain initiation and elongation of chondroitin/dermatan sulfate, one of the sulfated glycosaminoglycan chains that are covalently attached to various core proteins as proteoglycans.* SLC39A14* encodes the zinc transporter ZIP14, which is important for the intracellular Zn status and as such is comparable to ZIP13, previously shown to be mutated in the spondylocheirodysplastic form of EDS [8, 9]. Investigation of these genes with Sanger sequencing did not result in the identification of pathogenic alterations segregating with the phenotype.

In a next step, WES was performed for two affected sisters. The applied variant filtering strategy narrowed the number of shared heterozygous alterations down to a single missense variant in the* LZTS1* gene which was found to segregate with the disease phenotype.* LZTS1* is a tumor suppressor gene implicated in several human cancers. It is expressed in different normal tissues (Supplementary Figure S2) and was shown to be involved in cell cycle regulation through prevention of Cdc25C degradation thereby regulating Cdk1 activity [23]. In addition to its role in cancer,* LZTS1* was also shown to be important during neuronal development [24].* LZTS1* belongs, together with LZTS2, LZTS3, and N4BP, to the Fezzins, a family of proteins that share a leucine zipper Fez domain [25].* LZTS1* itself was not linked to joint hyperlaxity, but disruption of the ProSAP2 gene, encoding a protein interacting with LZTS2 (LAPSER1), resulted in a syndrome with mild intellectual disability and joint laxity [26, 27]. In an attempt to verify* LZTS1* as an EDS-HT predisposition gene, it was screened in 230 unrelated probands with EDS-HT, either evaluated at our connective tissue clinic or referred by another clinical geneticist. This revealed the presence of three additional* LZTS1* variants. Nevertheless, the contribution of these variants to the EDS-HT phenotype still remains unclear. It has been previously reported that the* LZTS1* gene and its protein contain features resembling those of transcription factors, which makes it tempting to speculate that* LZTS1* could have a role in modulating gene transcription and thereby affect the expression of genes encoding connective tissue molecules [28]. At present, however, we have no conclusive evidence for an exact function of* LZTS1* in connective tissue biology and further studies are necessary to confirm the pathogenic role of the identified sequence alterations. If a functional link could be established, the phenotypic discrepancy between patients with* LZTS1* defects within the same family could be attributed to incomplete penetrance and/or variable expressivity. Our results already indicate that* LZTS1* mutations will likely account for only a small proportion (around 2% as deduced from our results) of EDS-HT patients.

It should also be noted that, as the majority of disease-causing mutations reside within the protein-coding part of the genome, our study focused on the exonic part of the candidate interval, thereby omitting the interrogation of important noncoding regions, such as (deep) intronic regions, promoter sequences, and other regulatory and conserved sequences that reside within the candidate interval and which can also contain disease-causing alterations. In addition, the WES technology is also subjected to other limitations, such as the lack of a (small) proportion of genes/exons on the enrichment platform as well as the inefficient capturing and/or incomplete sequencing of some regions. Collectively, these issues can lead to false negative results and can mask the true underlying cause.

Efforts towards the identification of genes underlying joint hypermobility syndromes such as EDS-HT and BJHS are important in order to gain better insights into the clinical phenotypes, their natural history, and the underlying pathogenic basis. Families with clear autosomal dominant inheritance of EDS-HT probably represent rare forms of the condition, but elucidation of the causal gene defect in these families may allow identification of new genes and/or genetic pathways that are involved in EDS and joint hypermobility. This will improve early recognition and diagnosis of joint hypermobility and lead to a more logical classification of the hypermobility syndromes. For patients who are suffering from these chronic and painful conditions, accurate clinical diagnosis, confirmation of diagnosis by biochemical and/or molecular testing, better understanding of the pathogenic basis, and well-adjusted therapeutic follow-up by medical and paramedical staff are important factors that will help them cope with this condition and prevent feelings of frustration and depression. Understanding the genetic basis of joint hypermobility syndromes will moreover enhance our understanding of normal connective tissue biology and homeostasis and of mechanisms underlying EDS and joint mobility.

## Supplementary Material

Supplementary Table 1: Candidate genes, their chromosome localization and markers that were used for linkage analysis.
Supplementary Figure S1: Overview of the candidate region.
Supplementary Figure S2: RT-PCR based LZTS1 expression in human tissues.

## Figures and Tables

**Table 1:** Clinical characteristics of affected and unaffected family members.

	Age^*∗*^ (years)	Beighton score (/9)	Arthralgia		(Sub) luxation	Cutaneous					Tendonitis, muscle cramps	Other	Status
			CMPS >4 joints	CMPS 1–3 joints		Easy bruising	Soft skin	Skin hyperextensibility	Atrophic scarring/dilated scars	Striae			
I.2	75	5	+		Hip, TMJ, and sternoclavicular joint	+	+	−	+	−		Hernia umbilicalis, postoperative haemorrhages, and fragility of internal organs	A

II.1	47	2		+	Ankles	−	−	−	+	+	−		U

II.2	46	0	−	−		−	−	−	−	−	−		NA

II.3	45	6	+			+	+	−	−	+	+	Osteopenia, keratoconus, and muscle ruptures	A

II.4	44	5	+		Fingers, TMJ	+	+	+	+	+	+	Discus hernia	A

II.5	43	9	+		Generalized	+	+	−	+	+	+		A

II.6	42	5	+		Generalized	+	+	−	−	+	+		A

II.7	36	3	+		Shoulders, wrists, and patella	+	+	+	+	+	+		A

II.8	34	2	−			−	+/−	−	−	+	−		U

II.9	32	3	−	−	Shoulder	−	−	−	++	+	−		U

III.1	24	0	−	−		−	−	−	−	−	−		NA

III.2	19	0	−	−		−	−	−	−	−	−		NA

III.3	19	0	−	−		−	−	−	−	−	−		NA

III.4	17	2	−	+	Patella	+	−	−	−	−	−		U

III.5	15	2	−	−		−	−	−	−	−	−		NA

III.6	14	7	−		Fingers, ankles	+	+	+	+	−	−		A

III.7	10	2	−			−	−	−	−	−	−		NA

III.8	9	9	+		Wrist, toes	+	+	+	−	−	−	Transparent skin	A

III.9	6	2	−			−	−	−	−	−	−		NA

III.10	18	9	+		Fingers, patella	+	+	−	+	+	+		A

III.11	11	6	+			+	+	−	−	−	−		A

III.12	9	7	+			+	+	−	−	−	−		A

III.13	7	2	−	−		+/−	+/−	−	−	−	−		U

III.14	11	9	+		Ankles	+	+	+	−	−	−		A

III.15	11	2	−			−	−	−	−	−	−		NA

III.16	7	6	−	−		−	+	−	+	−	−		A

CMSP: chronic musculoskeletal pain for >3 months.

TMJ: temporomandibular joint.

A: “affected,” NA: “not affected,” and U: “unknown.”

^*∗*^Age at last clinical evaluation.

**Table 2:** Two-point LOD scores for microsatellite markers on the 8p22-8p21.1 region for a given *θ*.^*∗*^The identified candidate interval.

Marker	0	0.05	0.1	0.15	0.2	0.25	0.3	0.35	0.4	0.45
D8S254^*∗*^	0.21	*2.52*	2.49	2.32	2.08	1.79	1.46	1.10	0.71	0.34
D8S261^*∗*^	*1.86*	1.73	1.59	1.43	1.27	1.10	0.91	0.71	0.49	0.26
D8S258^*∗*^	**4.73**	4.35	3.95	3.53	3.09	2.62	2.12	1.58	1.02	0.47
D8S1771^*∗*^	2.04	*3.36*	3.26	3.01	2.69	2.32	1.89	1.43	0.92	0.42
D8S1820	−2.05	1.20	*1.29*	1.24	1.12	0.96	0.78	0.57	0.35	0.15

**Table 3:** Ranking of the candidate genes according to different gene prioritization tools with functionally interesting candidates indicated in bold.

	Endeavour^*∗*^	Suspects	G2D	GeneDistiller	ToppGene
1	***BMP1***	***BMP1***	*CNOT7*	***BMP1***	***CSGALNACT1***
2	***LOXL2***	*LPL*	***CSGALNACT1***	***CSGALNACT1***	*LPL*
3	***SLC39A14***	***CSGALNACT1***	*HR*	*NPM2*	***BMP1***
4	*DOCK5*	***SLC39A14***	*ADAMDEC1*	***LOXL2***	***LOXL2***
5	*LPL*	*ADAM7*	*GNRH1*	*LPL*	*GNRH1*
6	*STC1*	*ADAM28*	***BMP1***	*STC1*	*FGF17*
7	*SORBS3*	*ADAMDEC1*	*NKX3-1*	***SLC39A14***	*SFTPC*
8	*NEFL*	***LOXL2***	*STC1*	*GNRH1*	*FGF20*
9	*ADAM28*	*FGL1*	***SLC39A14***	*HR*	*HR*
10	*ASAH1*	*PDLIM2*	*LPL*	*GFRA2*	*STC1*
11	*CCAR2*	*LZTS1*	*NKX2-6*	*NAT1*	*DOK2*
12	*FGL1*	*FGF17*	*NAT1*	*TNFRSF10B*	*DMTN*
13	*EGF17*	*KTCD9*	*NPM2*	*NKX3-1*	*PDLIM2*
14	*ENTPP4*	*SLC18A1*	*ENTPD4*	*SFTPC*	*ATP6V1B2*
15	*PDGFRL*	*PCM1*	*ADAM28*	*PDLIM2*	*NPM2*
16	*PFLIM2*	*DOCK5*	*ASAH1*	*TNFRSF10D*	*FGL1*
17	*LZTS1*	*GFRA2*	*FGF17*	*SORBS3*	*NEFM*
18	*NAT1*	*SLC7A1*	*BIN3*	*POLR3D*	***SLC39A14***
19	*VSP37A*	*C8orf20*	***LOXL2***	*ASAH1*	*GFRA2*
20	*DOK2*	*STC1*	*ATP6V1B2*	*DOCK5*	*NEFL*

^*∗*^ *CSGALNACT1* is not recognized by the Endeavour web tool.

**Table 4:** Overview of the *LZTS1* variants identified in patients with EDS-HT or BJHS.

cDNA	Protein	SIFT	PolyPhen-2	Align	Mutation
				GVGD	Taster
c.633C>A	p.(His211Gln)	Tolerated	Probably damaging	C0	Disease-causing
c.49C>G	p.(His17Asp)	Tolerated	Probably damaging	C0	Disease-causing
c.1585C>T	p.(Arg529Trp)	Deleterious	Probably damaging	C0	Disease-causing
c.749C>A	p.(Ser250^*∗*^)	—	—	—	—
