# Supplementary material for: Ehlers-Danlos Syndrome, Hypermobility Type, Is Linked to Chromosome 8p22-8p21.1 in an Extended Belgian Family
Source: Dis Markers. 2015 Oct 4;2015:828970. doi: 10.1155/2015/828970 (PMC4609397; doi:10.1155/2015/828970)

SUPPLEMENTAL INFORMATION

Ehlers-Danlos syndrome, hypermobility type is linked to chromosome 8p22-8p21.1 in an extended Belgian family

Delfien Syx, Sofie Symoens, Wouter Steyaert, Anne De Paepe, Paul Coucke, Fransiska Malfait

Center for Medical Genetics, Ghent University Hospital, Ghent, Belgium

Supplementary Table 1: Candidate genes, their chromosome localization and markers that were used for linkage analysis.

Gene	Chromosome localization	Marker
COL1A1	17q21.3-q22	*D17S1795*<br>*D17S1820*
COL1A2	7q21.3-q22	*D7S527*<br>*D7S657*<br>*D7S1820*
COL3A1; COL5A2	2q32.2; 2q24.3-31	*D2S2262*<br>*D2S389*<br>*D2S118*
COL5A1	9q34.2-q34.3	*D9S1818*<br>*D9S1826*<br>*D9S1793*<br>*D9S67*<br>*D9S114*<br>*D9S158*
TNXB	6p21.3	*D6S1014*<br>*D6S2443*

Supplementary Figure S1: Overview of the candidate region. Printscreen from UCSC Genome Browser (https://genome.ucsc.edu).

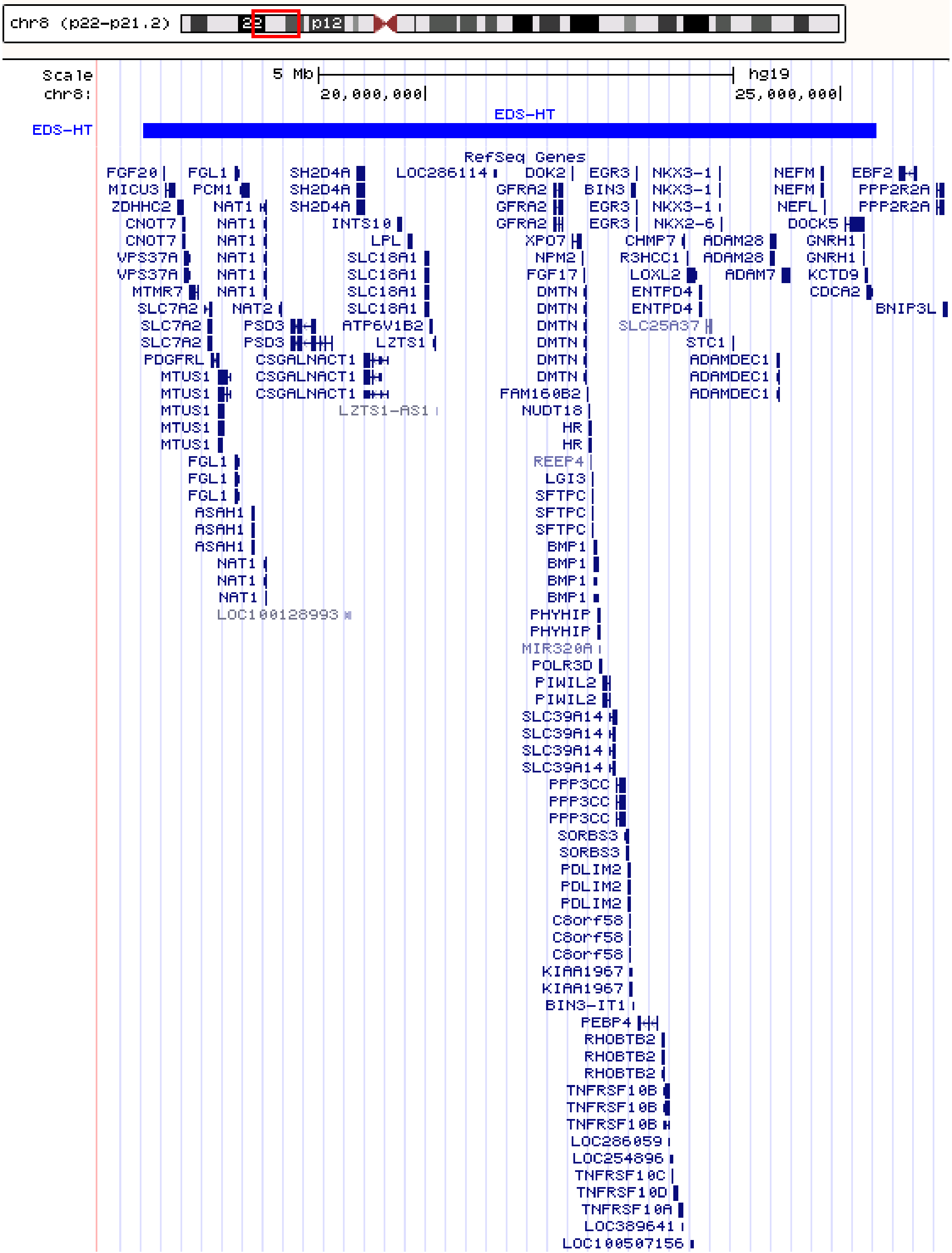

Supplementary Figure S2: RT-PCR based *LZTS1* expression in human tissues confirmed the highest *LZTS1* expression in brain and testes.

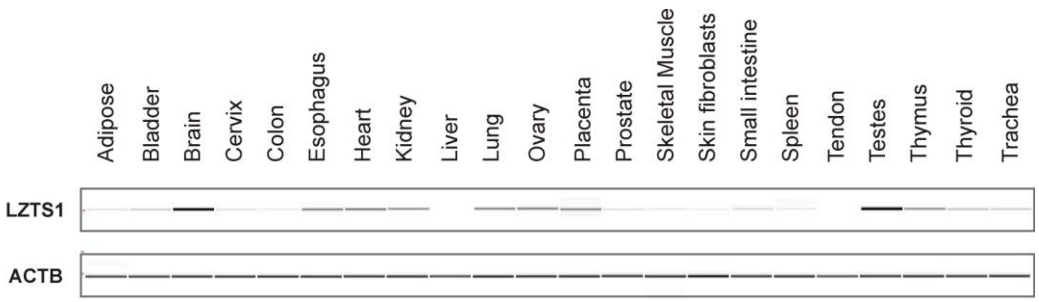

Supplement: Supplementary file 1 — Supplementary Table 1: Candidate genes, their chromosome localization and markers that were used for linkage analysis. Supplementary Figure S1: Overview of the candidate region. Supplementary Figure S2: RT-PCR based LZTS1 expression in human tissues. [file 828970.f1.pdf]
